# Risk factor analysis and prediction of lower-extremity deep vein thrombosis after surgery in patients with non-small cell lung cancer

**DOI:** 10.3389/fonc.2026.1874882

**Published:** 2026-07-30

**Authors:** Lan Zhuge, Qin Zhang

**Affiliations:** Department of Oncology, Jiaxing Hospital of Traditional Chinese Medicine Affiliated to Jiaxing University, Jiaxing, Zhejiang, China

**Keywords:** deep vein thrombosis, nomogram, non–small cell lung cancer, risk stratification, thromboprophylaxis

## Abstract

**Background:**

Postoperative lower-extremity deep vein thrombosis (DVT) remains a clinically relevant complication after surgical resection for non-small cell lung cancer (NSCLC). This study aimed to identify factors associated with postoperative DVT and develop a nomogram for estimating DVT probability in patients undergoing NSCLC resection.

**Methods:**

This retrospective cohort study included 328 patients with histologically confirmed NSCLC who underwent curative-intent pulmonary resection between January 2021 and December 2025. All patients underwent preoperative and postoperative lower-extremity duplex ultrasonography. The primary outcome was newly detected lower-extremity DVT within 30 days after surgery. Univariable and multivariable logistic regression analyses were performed to evaluate variables associated with postoperative DVT. A nomogram was developed, and model performance was assessed using receiver operating characteristic (ROC) analysis, bootstrap internal validation, and calibration analysis.

**Results:**

Among the included patients, 136 were assigned to the postoperative DVT group and 192 to the non-DVT group. Most DVT events were diagnosed during the index hospitalization and were asymptomatic, isolated distal DVT. In the primary multivariable model, age ≥70 years (OR, 4.50; 95% CI, 1.21–17.85), diabetes mellitus (OR, 5.00; 95% CI, 1.16–22.55), adenocarcinoma histology (OR, 7.55; 95% CI, 1.80–30.44), and pathological stage III disease (OR, 5.16; 95% CI, 1.39–19.05) were independently associated with postoperative DVT. The nomogram showed an apparent area under the curve (AUC) of 0.896, with sensitivity of 86.15% and specificity of 91.25%. The bootstrap-corrected concordance index was 0.762, and the Hosmer–Lemeshow test showed no evidence of poor fit.

**Conclusions:**

Age, diabetes mellitus, adenocarcinoma histology, and pathological stage III disease were associated with postoperative DVT in patients with resected NSCLC. The nomogram might support postoperative DVT risk estimation, but further external validation is warranted.

## 1 Introduction

Early postoperative deep vein thrombosis (DVT) of the lower extremities represents a significant complication in patients undergoing surgical resection for non-small cell lung cancer (NSCLC) (1). NSCLC accounts for approximately 85% of all lung malignancies and remains a leading cause of cancer-related mortality worldwide (2). Surgical intervention, encompassing lobectomy, bilobectomy, pneumonectomy, or segmentectomy, constitutes the primary curative approach for early-stage disease (3). However, the surgical stress response, prolonged immobilization, and malignancy-associated hypercoagulability converge to elevate the risk of venous thromboembolism (VTE), of which DVT is a cardinal manifestation (4, 5). The pathogenesis of postoperative DVT in NSCLC patients is multifactorial and aligns with Virchow’s triad, comprising venous stasis, endothelial injury, and hypercoagulability. Perioperative immobilization diminishes calf muscle pump function, while vessel wall trauma incurred during lymphadenectomy or pulmonary vessel ligation precipitates endothelial disruption (6, 7). Concurrently, tumor-derived procoagulant factors, including tissue factor–bearing microparticles and cancer procoagulant proteases, amplify thrombin generation (8). Systemic inflammatory mediators, such as interleukin-6 and C-reactive protein, further potentiate platelet activation and fibrin deposition (9, 10).

Clinical and laboratory parameters proposed as DVT risk factors in NSCLC encompass advanced age, elevated body mass index, a history of tobacco exposure, and comorbidities such as chronic obstructive pulmonary disease or diabetes mellitus (11). Operative variables, namely extended anesthesia duration, extensive lymph node dissection, and intraoperative blood loss, have likewise been implicated (12). Biomarkers including D-dimer, platelet count, and neutrophil-to-lymphocyte ratio offer adjunctive prognostic value, yet their standalone predictive accuracy remains suboptimal (13). Predictive modeling, particularly nomogram-based approaches, has emerged as a practical tool to quantify individualized DVT risk and guide perioperative management (14). By integrating quantitative coefficients derived from multivariable logistic regression, nomograms translate complex statistical relationships into user-friendly graphical scales. Such models have demonstrated favorable discrimination and calibration in other oncologic surgical cohorts, yet their application in NSCLC remains underexplored (15–17).

This study aims to identify independent risk factors for lower extremity DVT following NSCLC surgery and to construct a nomogram-based model for individualized risk prediction. Through retrospective analysis of perioperative clinical, surgical, and laboratory data, we endeavor to deliver a validated tool capable of informing prophylactic strategies and optimizing patient outcomes.

## 2 Methods

### 2.1 Study design

A retrospective cohort of patients with histologically confirmed non–small cell lung cancer who underwent surgical resection at our institution between January 2021 and December 2025 was assembled, comprising 328 eligible individuals. Postoperative development of lower extremity DVT served as the basis for stratification into a DVT group (n=136) and a non-DVT group (n=192). The study was conducted following the STROBE (Strengthening the Reporting of Observational Studies in Epidemiology) guidelines (18). Informed consent was obtained from all subjects and/or their legal guardian(s). The study was reviewed and approved by the hospital’s ethics committee. All procedures complied with relevant guidelines and regulations, adhering to the ethical principles of the Declaration of Helsinki. Participant data were anonymized prior to analysis to ensure confidentiality and privacy.

### 2.2 Inclusion and exclusion criteria

#### 2.2.1 Inclusion criteria

1. Histologically confirmed non–small cell lung cancer (NSCLC), clinical stage I–III.
2. Age ≥ 18 years at the time of surgery.
3. Underwent curative-intent pulmonary resection (lobectomy, bilobectomy, pneumonectomy, or segmentectomy) at our institution between January 2021 and December 2025.
4. No evidence of preoperative lower-extremity deep vein thrombosis on duplex ultrasonography.
5. Availability of complete perioperative clinical, surgical, and laboratory data, including postoperative lower-extremity venous imaging.

#### 2.2.2 Exclusion criteria

1. History of venous thromboembolism (deep vein thrombosis or pulmonary embolism) prior to the index surgery.
2. Preexisting coagulation disorders or ongoing therapeutic anticoagulation at the time of surgery.
3. Radiologically or pathologically confirmed distant metastases (stage IV disease).
4. Concurrent active malignancy other than NSCLC.
5. Incomplete medical records or loss to follow-up within 30 days postoperatively.
6. Perioperative mortality (death occurring intraoperatively or within 30 days of surgery).

### 2.3 Clinical, surgical, laboratory, and thromboprophylaxis data collection

Demographic, clinical, tumor-related, surgical, laboratory, hospitalization, and thromboprophylaxis-related variables were extracted from the electronic medical records. Demographic and clinical variables included age at surgery, sex, body mass index (BMI), smoking status, diabetes mellitus, hypertension, hyperlipidemia, chronic obstructive pulmonary disease (COPD), and Eastern Cooperative Oncology Group (ECOG) performance status. Smoking status was categorized as never smoking versus former/current smoking. ECOG performance status was dichotomized as 0–1 versus ≥2.

Tumor-related variables included histological subtype, pathological TNM stage, and receipt of neoadjuvant chemotherapy. Histology was categorized as adenocarcinoma versus non-adenocarcinoma. Pathological stage was categorized as stage I–II versus stage III according to postoperative pathological assessment. Surgical variables included surgical approach, type of pulmonary resection, surgery duration, estimated intraoperative blood loss, postoperative complications, and delayed first ambulation or bed-bound status. Surgical approach was categorized as open thoracotomy versus thoracoscopic surgery. Type of pulmonary resection was categorized as lobectomy, segmentectomy, bilobectomy, or pneumonectomy. Postoperative complications included clinically documented pulmonary infection, atelectasis requiring intervention, prolonged air leak, postoperative bleeding, wound infection, or other complications occurring before DVT diagnosis.

Preoperative laboratory data were collected within 48 hours before surgery. Laboratory variables included serum albumin, white blood cell count, platelet count, hemoglobin, D-dimer, prothrombin time (PT), international normalized ratio (INR), activated partial thromboplastin time (APTT), and fibrinogen. Albumin was analyzed as a continuous variable and was also dichotomized as <35 g/L versus ≥35 g/L. D-dimer was analyzed as elevated versus normal according to the upper limit of the institutional reference range.

Length of postoperative hospitalization was defined as the interval from the date of surgery to the date of discharge. This variable was collected to contextualize the timing of DVT diagnosis but was not included in the primary prediction model because it may be influenced by postoperative DVT occurrence.

### 2.4 DVT screening, outcome definition, and anatomical classification

All eligible patients underwent preoperative bilateral lower-extremity duplex ultrasonography to exclude preexisting lower-extremity DVT. Postoperative bilateral lower-extremity duplex ultrasonography was routinely performed regardless of symptoms, usually between postoperative days 3 and 7 or before hospital discharge. Additional symptom-driven ultrasonography was performed during the 30-day postoperative observation period if patients developed symptoms suggestive of DVT, including lower-extremity swelling, pain, tenderness, skin temperature change, or unexplained clinical suspicion of venous thrombosis.

The primary outcome was newly detected lower-extremity DVT confirmed by duplex ultrasonography within 30 days after surgery. DVT diagnosed beyond 30 days after surgery was not included in the primary outcome. Both symptomatic and asymptomatic DVT events were included. Asymptomatic DVT was defined as thrombosis detected by routine screening ultrasonography in patients without clinical symptoms suggestive of DVT.

DVT was classified according to anatomical location as proximal DVT, non-intermuscular distal DVT, or intermuscular calf vein thrombosis. Proximal DVT included thrombosis involving the iliac, femoral, or popliteal veins. Non-intermuscular distal DVT included thrombosis involving the posterior tibial, anterior tibial, or peroneal veins. Intermuscular calf vein thrombosis included thrombosis involving the gastrocnemius or soleal venous plexus. When thrombosis involved both proximal and distal veins, it was classified as proximal DVT for descriptive analysis.

The timing of DVT diagnosis was calculated as the number of days from surgery to the first positive postoperative ultrasonographic examination. For patients who developed DVT, only the first postoperative DVT event was included in the primary analysis.

### 2.5 Thromboprophylaxis protocol and exposure assessment

Postoperative thromboprophylaxis was implemented according to institutional practice and individual clinical assessment of thrombotic and bleeding risks. Thromboprophylaxis-related variables included low-molecular-weight heparin (LMWH) use, overall pharmacologic thromboprophylaxis, duration of pharmacologic thromboprophylaxis, and mechanical thromboprophylaxis.

Pharmacologic thromboprophylaxis was defined as the postoperative use of LMWH or other anticoagulant agents for venous thromboembolism prevention. LMWH use was recorded separately because it was the predominant pharmacologic prophylaxis strategy in this cohort. Aspirin prescribed solely for cardiovascular indications was not considered pharmacologic thromboprophylaxis unless it was explicitly documented as part of the postoperative DVT prophylaxis regimen. Mechanical thromboprophylaxis included intermittent pneumatic compression and/or graduated compression stockings.

For patients who developed postoperative DVT, thromboprophylaxis-related exposures, postoperative complications, and ambulation-related variables were considered only if documented before the diagnosis of DVT. For patients without DVT, these exposures were assessed during the corresponding 30-day postoperative observation period.

### 2.6 Statistical analysis

Statistical analyses were performed using SPSS version 29.0 and R version 4.3.3. SPSS was used for descriptive statistics, between-group comparisons, and logistic regression analyses, whereas R was used for nomogram construction, receiver operating characteristic (ROC) analysis, bootstrap validation, and calibration plotting. Continuous variables were summarized as mean ± standard deviation or median with interquartile range, as appropriate, and categorical variables were expressed as frequencies and percentages. Between-group comparisons were performed using the independent-samples t test or Mann–Whitney U test for continuous variables and the chi-square test or Fisher’s exact test for categorical variables. Univariable logistic regression was used to evaluate candidate variables associated with postoperative lower-extremity DVT. Variables with statistical significance in univariable analysis and clinically relevant thrombotic-risk covariates were considered for multivariable modeling. A primary multivariable logistic regression model was constructed for nomogram development. Sensitivity analyses were performed after excluding isolated intermuscular calf vein thrombosis and after additional adjustment for selected clinically relevant covariates. For the clinically adjusted sensitivity model, the number of covariates was restricted to maintain an acceptable events-per-variable (EPV) ratio. Multicollinearity among included variables was assessed using the variance inflation factor (VIF), with VIF <5 indicating no severe multicollinearity. The nomogram was constructed using the “rms” package in R. Discrimination was assessed using ROC analysis and the area under the curve (AUC). Internal validation was performed with 1, 000 bootstrap resamples. Calibration was evaluated using the Hosmer–Lemeshow goodness-of-fit test and calibration plot. All tests were two-sided, and P < 0.05 was considered statistically significant.

## 3 Results

### 3.1 Baseline and perioperative characteristics

A total of 328 patients with NSCLC were included, comprising 136 patients with postoperative lower-extremity DVT and 192 patients without DVT. Baseline demographic, clinical, tumor-related, perioperative, hospitalization, laboratory, and thromboprophylaxis-related characteristics are summarized in **Table 1**. Compared with the non-DVT group, the DVT group had higher proportions of patients aged ≥70 years, diabetes mellitus, hypertension, hyperlipidemia, and ECOG performance status ≥2. Former or current smoking and COPD showed borderline between-group differences, whereas sex and BMI category were not significantly different between groups. Tumor-related characteristics differed between groups. Adenocarcinoma histology, pathological stage III disease, and neoadjuvant chemotherapy were more frequently observed in the DVT group. Regarding perioperative and hospitalization variables, open thoracotomy, surgery duration ≥2 h, higher estimated blood loss, estimated blood loss ≥200 mL, postoperative complications, and longer postoperative hospitalization were more frequently observed in the DVT group. Delayed first ambulation or bed-bound status showed a borderline difference. For laboratory variables, the DVT group showed lower albumin and hemoglobin levels, higher platelet count, higher D-dimer levels, and a higher proportion of elevated D-dimer. Significant between-group differences were also observed in WBC count, PT, INR, fibrinogen, and APTT. Thromboprophylaxis-related variables, including LMWH use, pharmacologic thromboprophylaxis, duration of pharmacologic thromboprophylaxis, and mechanical prophylaxis, were comparable between the two groups.

**Table 1:** Baseline, perioperative, hospitalization, laboratory, and thromboprophylaxis-related characteristics of NSCLC patients stratified by postoperative DVT status.

Variable	DVT group (n = 136)	Non-DVT group (n = 192)	P value
Demographic characteristics			
Age ≥70 years	126 (92.6)	104 (54.2)	<0.001
Male sex	87 (64.0)	106 (55.2)	0.112
BMI ≥25 kg/m²	26 (19.1)	35 (18.2)	0.839
Former/current smoking	62 (45.6)	68 (35.4)	0.064
Comorbidities and functional status			
Diabetes mellitus	63 (46.3)	25 (13.0)	<0.001
Hypertension	33 (24.3)	29 (15.1)	0.037
Hyperlipidemia	53 (39.0)	42 (21.9)	<0.001
COPD	22 (16.2)	18 (9.4)	0.064
ECOG performance status ≥2	36 (26.5)	18 (9.4)	<0.001
Tumor-related characteristics			
Adenocarcinoma histology	80 (58.8)	35 (18.2)	<0.001
Pathological stage III	117 (86.0)	77 (40.1)	<0.001
Neoadjuvant chemotherapy	52 (38.2)	29 (15.1)	<0.001
Perioperative and hospitalization variables			
Open thoracotomy	65 (47.8)	35 (18.2)	<0.001
Surgery duration ≥2 h	44 (32.4)	36 (18.8)	0.005
Estimated blood loss, mL	155 ± 37	142 ± 32	0.001
Estimated blood loss ≥200 mL	19 (14.0)	13 (6.8)	0.030
Delayed first ambulation or bed-bound status	57 (41.9)	63 (32.8)	0.092
Postoperative complications	30 (22.1)	20 (10.4)	0.004
Length of postoperative hospitalization, days	9 (7–12)	8 (6–10)	0.006
Laboratory variables			
Albumin, g/L	37.2 ± 4.8	39.4 ± 4.5	<0.001
Albumin <35 g/L	39 (28.7)	26 (13.5)	0.001
WBC, ×10^9^/L	7.19 ± 1.53	6.47 ± 1.39	<0.001
Platelet count, ×10^9^/L	268 ± 74	235 ± 68	<0.001
Hemoglobin, g/L	121 ± 16	128 ± 15	<0.001
D-dimer, mg/L FEU	1.28 (0.72–2.14)	0.76 (0.42–1.36)	<0.001
Elevated D-dimer	104 (76.5)	106 (55.2)	<0.001
PT, s	11.53 ± 0.21	11.27 ± 0.20	<0.001
INR	1.05 ± 0.09	1.01 ± 0.08	<0.001
Fibrinogen, g/L	3.35 ± 0.27	3.44 ± 0.18	0.001
APTT, s	31.95 ± 4.52	29.06 ± 3.65	<0.001
Thromboprophylaxis-related variables			
LMWH use	112 (82.4)	165 (85.9)	0.389
Pharmacologic thromboprophylaxis	118 (86.8)	173 (90.1)	0.346
Duration of pharmacologic thromboprophylaxis, days	5.7 ± 2.2	5.9 ± 2.1	0.409
Mechanical prophylaxis	131 (96.3)	185 (96.4)	0.988

NSCLC, non–small cell lung cancer; DVT, deep vein thrombosis; BMI, body mass index; COPD, chronic obstructive pulmonary disease; ECOG, Eastern Cooperative Oncology Group; WBC, white blood cell count; PT, prothrombin time; INR, international normalized ratio; APTT, activated partial thromboplastin time; FEU, fibrinogen-equivalent units.

### 3.2 Surgical details, thromboprophylaxis, and diagnostic characteristics of postoperative DVT

Surgical details, thromboprophylaxis-related variables, and diagnostic characteristics of postoperative DVT are summarized in **Table 2**. Open thoracotomy was more frequently observed in the DVT group, whereas the distribution of pulmonary resection type did not differ significantly between groups. Surgery duration ≥2 h, higher estimated blood loss, estimated blood loss ≥200 mL, postoperative complications, and longer postoperative hospitalization were more common in patients with postoperative DVT. Delayed first ambulation or bed-bound status showed a borderline between-group difference. Thromboprophylaxis-related variables were comparable between groups. No statistically significant differences were observed in LMWH use, overall pharmacologic thromboprophylaxis, duration of pharmacologic thromboprophylaxis, or mechanical prophylaxis. Among patients with postoperative DVT, most events were diagnosed during the index hospitalization, and no events were diagnosed beyond 30 days after surgery. The median time from surgery to DVT diagnosis was within the first postoperative week. Most DVT events were detected by routine screening ultrasonography and were asymptomatic at diagnosis. Regarding anatomical distribution, isolated distal DVT accounted for most postoperative DVT events, with intermuscular calf vein thrombosis being the most frequent subtype. Proximal DVT and non-intermuscular distal DVT accounted for a smaller proportion of cases. In the sensitivity analysis excluding isolated intermuscular calf vein thrombosis, age ≥70 years, diabetes mellitus, adenocarcinoma histology, and pathological stage III disease remained statistically associated with the restricted DVT outcome (**Supplementary Table 1**).

**Table 2:** Surgical details, thromboprophylaxis, and diagnostic characteristics of postoperative DVT.

Variable	DVT group (n = 136)	Non-DVT group (n = 192)	P value
Surgical approach			<0.001
Open thoracotomy	65 (47.8)	35 (18.2)	
Thoracoscopic surgery	71 (52.2)	157 (81.8)	
Type of pulmonary resection			0.109
Lobectomy	101 (74.3)	135 (70.3)	
Segmentectomy	18 (13.2)	43 (22.4)	
Bilobectomy	10 (7.4)	8 (4.2)	
Pneumonectomy	7 (5.1)	6 (3.1)	
Surgery duration ≥2 h	44 (32.4)	36 (18.8)	0.005
Estimated blood loss, mL	155 ± 37	142 ± 32	0.001
Estimated blood loss ≥200 mL	19 (14.0)	13 (6.8)	0.030
Delayed first ambulation or bed-bound status	57 (41.9)	63 (32.8)	0.092
Postoperative complications	30 (22.1)	20 (10.4)	0.004
Length of postoperative hospitalization, days	9 (7–12)	8 (6–10)	0.006
Thromboprophylaxis-related variables			
LMWH use	112 (82.4)	165 (85.9)	0.389
Pharmacologic thromboprophylaxis	118 (86.8)	173 (90.1)	0.346
Duration of pharmacologic thromboprophylaxis, days	5.7 ± 2.2	5.9 ± 2.1	0.409
Mechanical prophylaxis	131 (96.3)	185 (96.4)	0.988
Timing of DVT diagnosis	DVT patients (n = 136)		
During index hospitalization	129 (94.9)	—	
After discharge but within 30 days	7 (5.1)	—	
Beyond 30 days after surgery	0 (0.0)	—	
Time from surgery to DVT diagnosis, days	5 (3–7)	—	
Postoperative days 1–3	34 (25.0)	—	
Postoperative days 4–7	84 (61.8)	—	
Postoperative days 8–30	18 (13.2)	—	
Mode of detection	DVT patients (n = 136)		
Routine screening ultrasonography	112 (82.4)	—	
Symptom-driven ultrasonography	24 (17.6)	—	
Symptom status	DVT patients (n = 136)		
Asymptomatic DVT	112 (82.4)	—	
Symptomatic DVT	24 (17.6)	—	
Anatomical distribution	DVT patients (n = 136)		
Proximal DVT	14 (10.3)	—	
Isolated distal DVT	122 (89.7)	—	
Intermuscular calf vein thrombosis	94 (69.1)	—	
Non-intermuscular distal DVT	28 (20.6)	—	
Proximal or non-intermuscular distal DVT	42 (30.9)	—	

DVT, deep vein thrombosis.

### 3.3 Univariable analysis of factors associated with postoperative DVT

Univariable logistic regression was performed to evaluate candidate variables associated with postoperative lower-extremity DVT in patients with NSCLC (**Table 3**). Age ≥70 years, diabetes mellitus, hypertension, hyperlipidemia, ECOG performance status ≥2, albumin <35 g/L, adenocarcinoma histology, pathological stage III disease, neoadjuvant chemotherapy, open thoracotomy, surgery duration ≥2 h, estimated blood loss ≥200 mL, postoperative complications, and length of postoperative hospitalization were statistically associated with postoperative DVT. Among laboratory variables, WBC count, platelet count, hemoglobin, elevated D-dimer, PT, INR, and APTT were statistically associated with postoperative DVT in the univariable analysis, whereas fibrinogen was not significant. Former or current smoking, COPD, and delayed first ambulation or bed-bound status showed borderline associations. In contrast, sex, BMI category, pharmacologic thromboprophylaxis, duration of pharmacologic thromboprophylaxis, mechanical prophylaxis, and LMWH use were not significantly associated with postoperative DVT. Length of postoperative hospitalization was reported to contextualize the timing of DVT diagnosis and was not included in the primary prediction model because it may be influenced by postoperative DVT occurrence.

**Table 3:** Univariable analysis of factors associated with postoperative DVT in NSCLC patients.

Variable	Reference category or unit	Crude OR	95% CI	P value
Age ≥70 years	<70 years	10.66	5.27–21.55	<0.001
Male sex	Female	1.44	0.92–2.26	0.112
BMI ≥25 kg/m²	<25 kg/m²	1.06	0.60–1.86	0.839
Former/current smoking	Never smoking	1.53	0.98–2.39	0.064
Diabetes mellitus	No	5.76	3.36–9.88	<0.001
Hypertension	No	1.80	1.03–3.14	0.037
Hyperlipidemia	No	2.28	1.40–3.71	<0.001
COPD	No	1.87	0.96–3.63	0.064
ECOG performance status ≥2	0–1	3.48	1.88–6.45	<0.001
Albumin <35 g/L	≥35 g/L	2.57	1.47–4.48	0.001
WBC	Per 1 ×10^9^/L increase	1.38	1.18–1.61	<0.001
Platelet count	Per 100 ×10^9^/L increase	1.82	1.29–2.57	0.001
Hemoglobin	Per 10 g/L decrease	1.34	1.13–1.59	0.001
Elevated D-dimer	Normal D-dimer	2.63	1.60–4.33	<0.001
PT	Per 1-s increase	2.91	1.54–5.49	0.001
INR	Per 0.1-unit increase	1.58	1.22–2.04	<0.001
Fibrinogen	Per 1-g/L increase	0.78	0.52–1.17	0.231
APTT	Per 1-s increase	1.19	1.11–1.27	<0.001
Adenocarcinoma histology	Non-adenocarcinoma	6.41	3.88–10.57	<0.001
Pathological stage III	Stage I–II	9.20	5.23–16.17	<0.001
Neoadjuvant chemotherapy	No	3.48	2.06–5.88	<0.001
Open thoracotomy	Thoracoscopic surgery	4.11	2.50–6.75	<0.001
Surgery duration ≥2 h	<2 h	2.07	1.24–3.45	0.005
Estimated blood loss ≥200 mL	<200 mL	2.24	1.06–4.70	0.030
Delayed first ambulation or bed-bound status	No	1.48	0.94–2.33	0.092
Postoperative complications	No	2.43	1.32–4.50	0.004
Pharmacologic thromboprophylaxis	No	0.72	0.36–1.43	0.346
Duration of pharmacologic thromboprophylaxis	Per 1-day increase	0.95	0.84–1.07	0.409
Mechanical prophylaxis	No	0.99	0.31–3.19	0.988
LMWH use	No	0.77	0.39–1.52	0.389
Length of postoperative hospitalization	Per 1-day increase	1.10	1.02–1.18	0.006

NSCLC, non–small cell lung cancer; DVT, deep vein thrombosis; BMI, body mass index; COPD, chronic obstructive pulmonary disease; ECOG, Eastern Cooperative Oncology Group; WBC, white blood cell count; PT, prothrombin time; INR, international normalized ratio; APTT, activated partial thromboplastin time; LMWH, low-molecular-weight heparin; OR, odds ratio; CI, confidence interval.

Length of postoperative hospitalization was summarized to contextualize the timing of DVT diagnosis and was not included in the primary prediction model because it may be affected by postoperative DVT occurrence.

### 3.4 Multivariable logistic regression analysis

A primary multivariable logistic regression model was constructed to evaluate variables associated with postoperative lower-extremity DVT in patients with NSCLC. In this model, age ≥70 years, diabetes mellitus, adenocarcinoma histology, and pathological stage III disease were independently associated with postoperative DVT (**Table 4**).

**Table 4:** Primary multivariable logistic regression model for postoperative DVT in NSCLC patients.

Variable	β	SE	Wald χ²	Adjusted OR	95% CI	P value
Age ≥70 years	1.504	0.683	4.852	4.50	1.21–17.85	0.024
Diabetes mellitus	1.610	0.772	4.349	5.00	1.16–22.55	0.029
Adenocarcinoma histology	2.021	0.736	7.550	7.55	1.80–30.44	0.005
Pathological stage III	1.642	0.673	5.948	5.16	1.39–19.05	0.013

NSCLC, non–small cell lung cancer; DVT, deep vein thrombosis; β, regression coefficient; SE, standard error; OR, odds ratio; CI, confidence interval.

This table presents the primary parsimonious multivariable model used for nomogram construction. Additional clinically relevant covariates were evaluated in the sensitivity model shown in **Supplementary Table 2**.

To assess the robustness of the primary findings while reducing the risk of overfitting, a reduced clinically adjusted sensitivity model was further constructed. This model included the four variables retained in the primary model and selected clinically relevant covariates, including former/current smoking, COPD, ECOG performance status ≥2, albumin <35 g/L, elevated D-dimer, open thoracotomy, postoperative complications, delayed ambulation or bed-bound status, and pharmacologic thromboprophylaxis. The reduced sensitivity model included 13 variables and 136 postoperative DVT events, corresponding to an EPV of 10.5. In this model, age ≥70 years, diabetes mellitus, adenocarcinoma histology, and pathological stage III disease remained statistically associated with postoperative DVT, with adjusted effect estimates in the same direction as those observed in the primary model (**Supplementary Table 2**). The selected additional covariates were not statistically associated with postoperative DVT after adjustment.

Multicollinearity among variables included in the reduced sensitivity model was assessed using the VIF. No evidence of severe multicollinearity was observed, with VIF values ranging from 1.12 to 1.94 (**Supplementary Table 3**).

### 3.5 Nomogram construction

**Figure 1:**
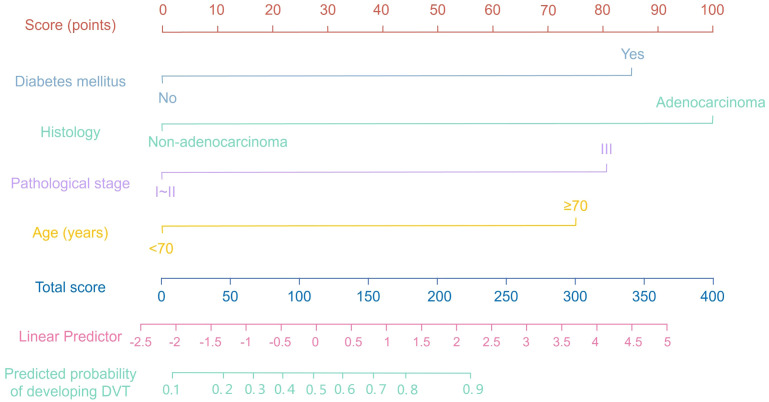
Nomogram for estimating the probability of postoperative lower-extremity DVT in patients undergoing resection for non-small cell lung cancer.

A nomogram for estimating the probability of postoperative lower-extremity DVT was constructed using the four variables retained in the primary multivariable logistic regression model: age ≥70 years, diabetes mellitus, adenocarcinoma histology, and pathological stage III disease. Each variable was assigned a weighted point value according to its regression coefficient, and the total score corresponded to an estimated probability of postoperative DVT on the nomogram scale (**Figure 1**).

### 3.6 Discriminative ability

**Figure 2:**
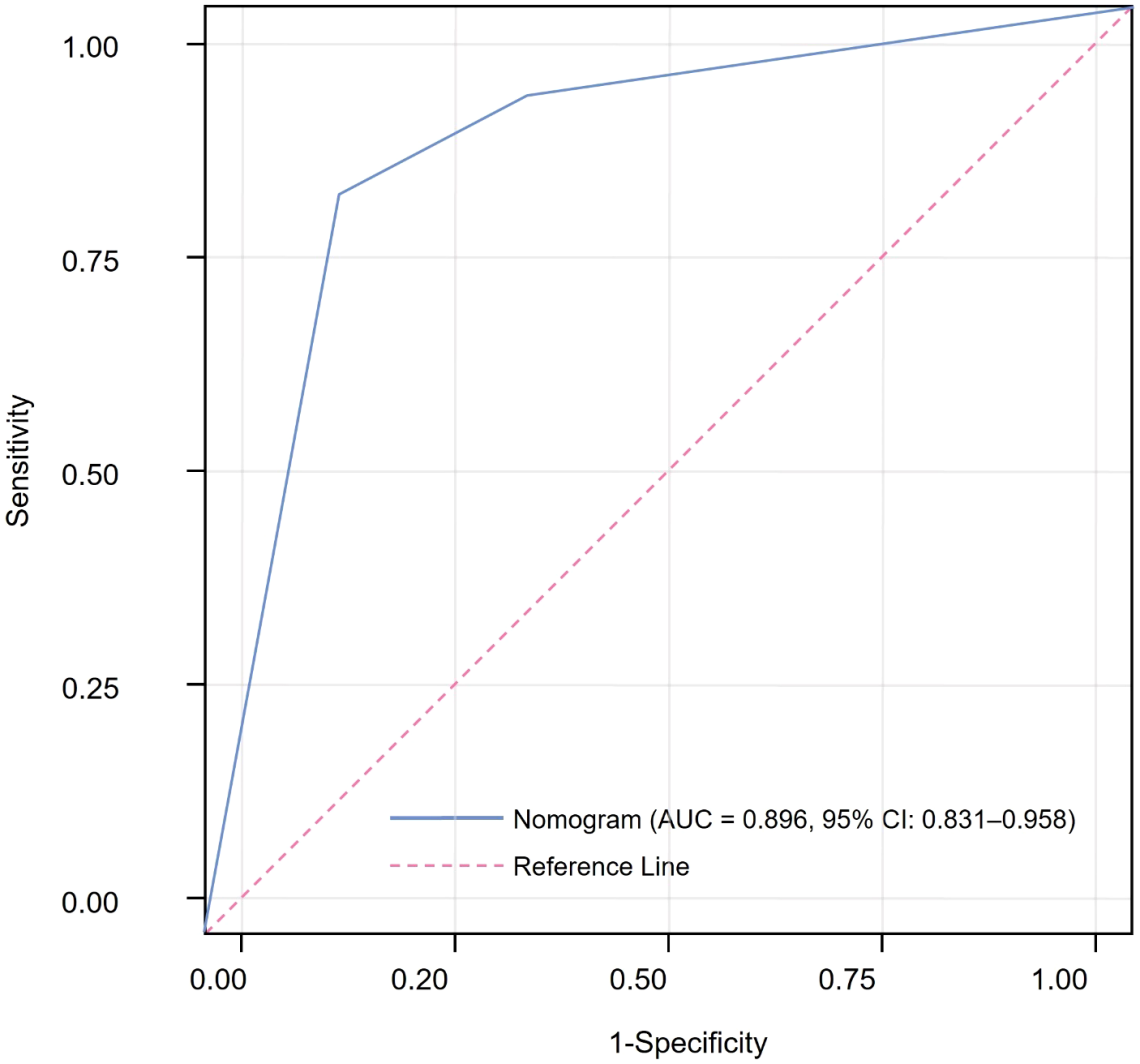
Receiver operating characteristic curve of the nomogram for postoperative lower-extremity DVT in patients with NSCLC.

The discriminative performance of the nomogram was evaluated using ROC curve analysis. The apparent AUC was 0.896 (95% CI, 0.831–0.958). At the optimal cutoff determined by the maximum Youden index, the sensitivity was 86.15% and the specificity was 91.25% (**Figure 2**).

### 3.7 Calibration and internal validation

**Figure 3:**
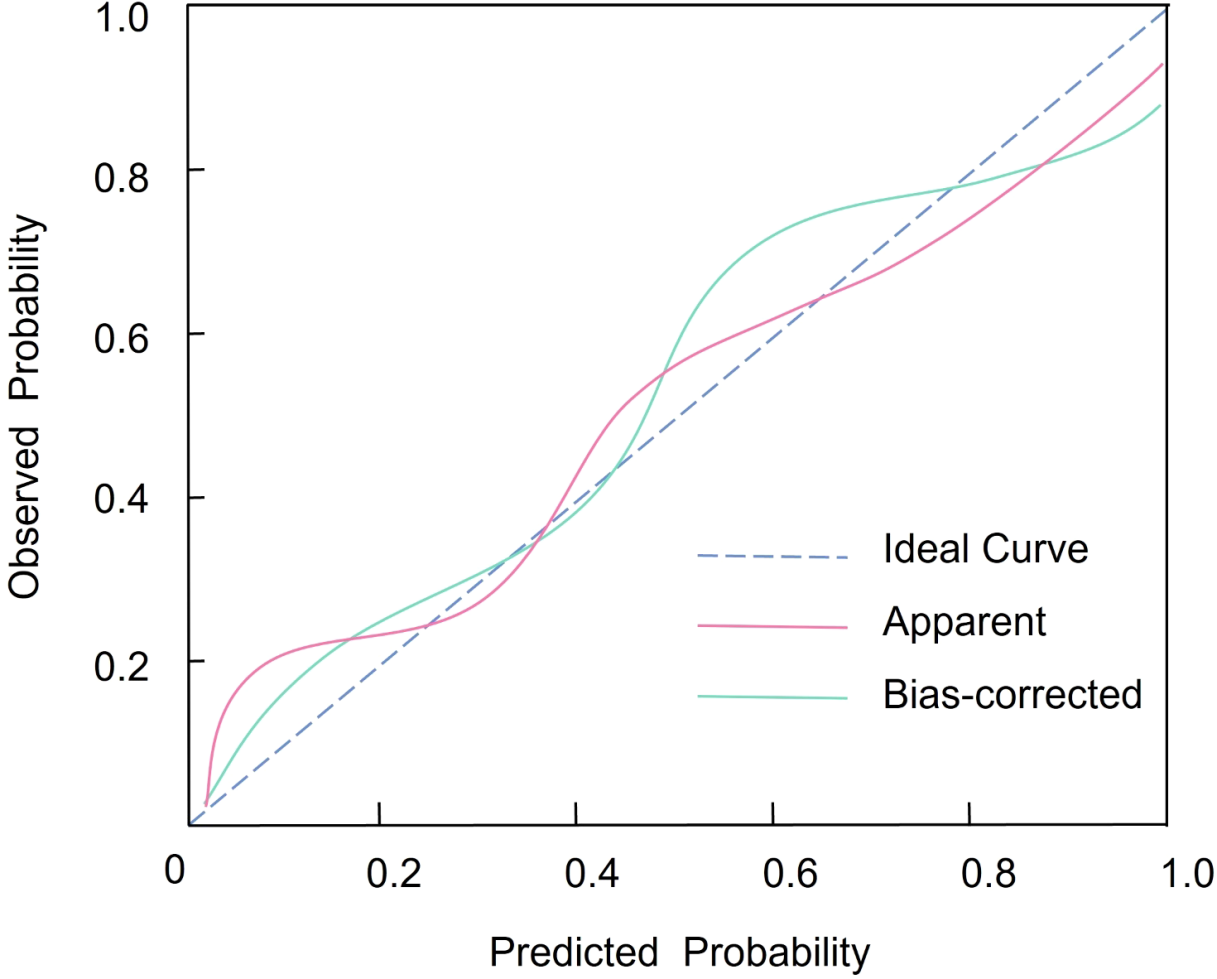
Calibration plot comparing nomogram-predicted probabilities with observed probabilities of postoperative lower-extremity DVT.

Internal validation was performed using 1, 000 bootstrap resamples, yielding a bias-corrected C-index of 0.762 (95% CI, 0.715–0.895). Calibration was assessed using the Hosmer–Lemeshow goodness-of-fit test and calibration plot. The Hosmer–Lemeshow test showed no statistically significant evidence of poor fit (χ² = 2.579, P = 0.852). The calibration plot showed agreement between predicted and observed postoperative DVT probabilities within the study cohort (**Figure 3**).

## 4 Discussion

In this retrospective cohort of patients with resected NSCLC, we systematically characterized early postoperative lower-extremity DVT using routine duplex ultrasonography, detailed diagnostic timing, anatomical classification, and perioperative thromboprophylaxis information. A notable feature of this study is that both symptomatic and asymptomatic events were captured, allowing a more comprehensive description of ultrasound-detected postoperative DVT, as reflected by the predominance of isolated distal and intermuscular calf vein thrombosis in this cohort (19). After evaluating demographic, tumor-related, surgical, laboratory, functional-status, and thromboprophylaxis-related variables, age ≥70 years, diabetes mellitus, adenocarcinoma histology, and pathological stage III disease were statistically associated with postoperative DVT in the primary model, with similar findings observed in the sensitivity analyses. The resulting nomogram showed discrimination and calibration performance within the study cohort and may provide a structured approach for postoperative DVT risk estimation. Because pathological stage is determined after surgery, this model should be interpreted primarily as a tool for postoperative risk reassessment rather than purely preoperative stratification (20, 21). Clinically, these findings may help identify patients who warrant closer postoperative surveillance, while any model-informed decisions regarding extended anticoagulation require prospective validation with assessment of bleeding risk and net clinical benefit (22, 23).

The observed postoperative DVT frequency should be interpreted in relation to how DVT was ascertained in this cohort. Because postoperative duplex ultrasonography was performed routinely during the early postoperative period, the study captured not only clinically apparent thrombotic events but also subclinical thrombosis that may have been missed in symptom-driven diagnostic pathways. This distinction is important when comparing DVT rates across thoracic surgery studies, as reported incidence is influenced by screening intensity, timing of imaging, and whether asymptomatic distal thrombosis is included in the endpoint definition. In the present cohort, most DVT events were identified during the index hospitalization, and the median time to diagnosis was within the first postoperative week. This temporal pattern suggests that the outcome mainly reflected early postoperative thrombosis rather than delayed venous thromboembolic events occurring later in follow-up. Anatomically, the majority of events were isolated distal DVT, particularly intermuscular calf vein thrombosis, whereas proximal DVT accounted for a relatively small proportion. These findings suggest that the high DVT frequency observed in this cohort was likely related to the screening-based outcome definition, early postoperative imaging, and inclusion of asymptomatic distal thrombosis (24). From a clinical perspective, this distribution emphasizes the need to distinguish between overall postoperative DVT burden and clinically overt or proximal events when interpreting risk estimates (25, 26). The early timing of detection also highlights the potential relevance of perioperative monitoring and risk reassessment before discharge, especially in patients with multiple clinical or tumor-related risk features (27, 28).

The factors retained in the primary multivariable model reflect complementary dimensions of postoperative thrombotic susceptibility in patients with NSCLC. Older age may indicate reduced physiological reserve, impaired venous return, higher comorbidity burden, and greater vulnerability to perioperative immobilization (29). Diabetes mellitus is closely linked to endothelial dysfunction, platelet activation, chronic low-grade inflammation, and metabolic abnormalities, all of which may contribute to a prothrombotic milieu (30, 31). The associations observed for adenocarcinoma histology and pathological stage III disease suggest that tumor-related characteristics also provide relevant information for postoperative DVT risk estimation (32). Lung adenocarcinoma has been frequently discussed in the context of cancer-associated thrombosis, potentially through tumor-driven coagulation activation and mucin-related pathways, whereas more advanced pathological stage may reflect greater tumor burden, more intensive treatment exposure, and heightened systemic inflammatory or hypercoagulable status (33, 34). In the univariable analysis, several surgical, functional, laboratory, and hospitalization-related variables were associated with DVT, including ECOG performance status, albumin, D-dimer, platelet count, WBC count, PT, INR, APTT, surgical approach, operative duration, blood loss, postoperative complications, and hospitalization length. In the reduced clinically adjusted sensitivity model, however, the selected additional covariates were not statistically associated with postoperative DVT after adjustment. The persistence of the four primary predictors across sensitivity analyses suggests the stability of these associations within the available dataset. In addition, the comparable use of pharmacologic and mechanical thromboprophylaxis between groups makes it less likely that the observed associations were mainly explained by measured differences in recorded prophylaxis exposure.

Our findings should also be interpreted in relation to recent studies evaluating postoperative thromboembolic risk after lung cancer surgery. Ji et al. (35) developed and externally validated a nomogram for postoperative DVT in patients with NSCLC and identified age ≥70 years, diabetes mellitus, adenocarcinoma histology, and stage III disease as independent predictors. These findings are highly consistent with our results, suggesting that these clinical and tumor-related factors may have stable relevance across NSCLC surgical cohorts. Compared with that study, our analysis further characterized diagnostic timing, symptom status, anatomical DVT distribution, perioperative thromboprophylaxis exposure, and sensitivity analyses excluding isolated intermuscular calf vein thrombosis. In addition to NSCLC-specific prediction studies, large database analyses have also highlighted the relevance of tumor stage and surgical complexity. Axtell et al. (36) reported that postoperative thromboembolic events after lung cancer resection were associated with locally advanced disease, greater surgical extent, longer operative duration, and thoracotomy, whereas minimally invasive surgery was associated with lower postoperative thromboembolic risk. Consistently, our univariable analysis showed associations of open thoracotomy, longer surgery duration, and stage III disease with postoperative DVT; however, after multivariable adjustment, mainly patient- and tumor-related variables remained associated with DVT. Lin et al. (37) further constructed a prediction model for postoperative VTE in elderly lung cancer patients and reported associations with high Caprini score, prolonged surgery, open surgery, advanced clinical stage, preoperative chemotherapy, elevated D-dimer, and C-reactive protein. Compared with their elderly-specific VTE model, our cohort included a broader NSCLC surgical population and focused specifically on lower-extremity DVT detected by routine ultrasonography. Taken together, these studies and our findings indicate that postoperative thrombotic risk assessment after lung cancer surgery may require integration of host characteristics, tumor burden, operative factors, and screening-defined outcome characteristics.

The clinical value of this study lies in its potential to support structured postoperative DVT risk assessment in patients undergoing resection for NSCLC. The nomogram includes four routinely available variables: age, diabetes mellitus, adenocarcinoma histology, and pathological stage. Because pathological stage III disease is determined after surgery, the nomogram should not be interpreted as a purely preoperative risk-stratification tool, but rather as a postoperative risk reassessment tool once pathological information is available. By translating these variables into an estimated DVT probability, the nomogram may help clinicians identify patients who warrant closer postoperative surveillance, careful assessment of lower-extremity symptoms, and timely duplex ultrasonography when clinically indicated. A representative application example is provided in **Supplementary Example 1**. The predominance of early, in-hospital, asymptomatic, and distal DVT events in this cohort further suggests that reassessment before discharge may be clinically meaningful. However, the nomogram should be regarded as a supportive risk-estimation tool rather than a standalone basis for modifying thromboprophylaxis. Decisions regarding pharmacologic prophylaxis, extended anticoagulation, or intensified monitoring should still consider bleeding risk, surgical recovery, comorbidities, and institutional protocols. Further external validation is needed before broader clinical implementation.

Several limitations should be acknowledged. The retrospective, single-center design introduces potential selection bias and limits external generalizability. Although we conducted internal validation through bootstrap resampling, external validation in diverse populations is essential before widespread adoption. Data on bleeding events were not systematically captured, precluding a formal net-clinical-benefit analysis of extended anticoagulation. Additionally, reliance on pathologic rather than clinical staging restricts the model’s use for purely preoperative decision making, though preoperative imaging and nodal sampling often approximate final pathologic stage. Finally, certain laboratory markers, such as D−dimer or pro-inflammatory cytokines, were unavailable for all patients and might further refine risk estimation if incorporated in future models. Despite these limitations, our study possesses notable strengths. The cohort was homogenous, comprising exclusively NSCLC surgery patients managed under standardized perioperative pathways, which reduces heterogeneity. The nomogram is parsimonious, requiring only four variables routinely assessed in clinical practice, yet it achieves superior discriminative performance compared to more complex models. Calibration analysis confirmed that predicted probabilities align closely with observed outcomes, indicating reliability in risk estimation within our cohort. Moreover, the model’s simplicity facilitates rapid bedside application without additional laboratory testing or specialized software. Future studies should aim to validate this nomogram in external cohorts, ideally through multicenter prospective designs that include broader patient demographics and varying prophylactic regimens. Incorporation of dynamic postoperative factors may enhance prediction accuracy. Additionally, randomized trials could assess whether nomogram-guided prophylaxis strategies improve clinical outcomes compared to standard protocols. Such efforts would establish whether individualized risk stratification translates into fewer thrombotic events, reduced hospitalization duration, and improved quality of life without unacceptable bleeding risk.

## 5 Conclusions

In conclusion, age ≥70 years, diabetes mellitus, adenocarcinoma histology, and pathological stage III disease were independently associated with postoperative lower-extremity DVT in patients with NSCLC. The nomogram based on these variables showed good discrimination and calibration within the study cohort and may support postoperative DVT risk estimation. Further external validation and prospective studies assessing clinical outcomes, bleeding risk, and net clinical benefit are needed before any model-based thromboprophylaxis strategy can be recommended.

## Data Availability

The raw data supporting the conclusions of this article will be made available by the authors, without undue reservation.
