# Supplementary material for: Risk factor analysis and prediction of lower-extremity deep vein thrombosis after surgery in patients with non-small cell lung cancer
Source: Front Oncol. 2026 Jul 30;16:1874882. doi: 10.3389/fonc.2026.1874882 (PMC13467825; doi:10.3389/fonc.2026.1874882)
Supplement: Supplementary file 1 [file Table1.docx]

Supplementary Table S1. Sensitivity analysis after excluding isolated intermuscular calf vein thrombosis.

| Variable | Adjusted OR | 95% CI | P value |
| --- | --- | --- | --- |
| Age ≥70 years | 3.02 | 1.12–8.14 | 0.029 |
| Diabetes mellitus | 2.85 | 1.22–6.65 | 0.016 |
| Adenocarcinoma histology | 2.96 | 1.35–6.47 | 0.007 |
| Pathological stage III | 3.21 | 1.46–7.04 | 0.004 |

Abbreviations: OR, odds ratio; CI, confidence interval; DVT, deep vein thrombosis.

Supplementary Table S2. Sensitivity analysis with selected clinically relevant covariates.

| Variable | Adjusted OR | 95% CI | P value |
| --- | --- | --- | --- |
| Age ≥70 years | 4.18 | 1.14–15.32 | 0.031 |
| Diabetes mellitus | 4.76 | 1.15–19.72 | 0.032 |
| Adenocarcinoma histology | 6.94 | 1.70–28.36 | 0.007 |
| Pathological stage III | 4.89 | 1.29–18.52 | 0.019 |
| Former/current smoking | 1.19 | 0.61–2.35 | 0.606 |
| COPD | 1.28 | 0.51–3.20 | 0.599 |
| ECOG performance status ≥2 | 1.44 | 0.62–3.36 | 0.396 |
| Albumin <35 g/L | 1.31 | 0.56–3.07 | 0.534 |
| Elevated D-dimer | 1.51 | 0.72–3.17 | 0.276 |
| Open thoracotomy | 1.63 | 0.72–3.68 | 0.241 |
| Postoperative complications | 1.37 | 0.58–3.24 | 0.474 |
| Delayed ambulation or bed-bound status | 1.32 | 0.65–2.67 | 0.440 |
| Pharmacologic thromboprophylaxis | 0.82 | 0.31–2.17 | 0.690 |

Abbreviations: NSCLC, non–small cell lung cancer; DVT, deep vein thrombosis; COPD, chronic obstructive pulmonary disease; ECOG, Eastern Cooperative Oncology Group; OR, odds ratio; CI, confidence interval.

Supplementary Table S3. Multicollinearity assessment for variables included in the reduced clinically adjusted sensitivity model.

| Variable | VIF |
| --- | --- |
| Age ≥70 years | 1.42 |
| Diabetes mellitus | 1.31 |
| Adenocarcinoma histology | 1.57 |
| Pathological stage III | 1.94 |
| Former/current smoking | 1.38 |
| COPD | 1.35 |
| ECOG performance status ≥2 | 1.62 |
| Albumin <35 g/L | 1.54 |
| Elevated D-dimer | 1.46 |
| Open thoracotomy | 1.87 |
| Postoperative complications | 1.72 |
| Delayed ambulation or bed-bound status | 1.49 |
| Pharmacologic thromboprophylaxis | 1.12 |

Abbreviations: VIF, variance inflation factor; COPD, chronic obstructive pulmonary disease; ECOG, Eastern Cooperative Oncology Group.

Supplementary Example 1. Example of nomogram application for estimating postoperative DVT probability

To illustrate the application of the nomogram, we considered a 69-year-old patient with resected non-small cell lung cancer. The patient had no history of diabetes mellitus. Postoperative pathological assessment confirmed adenocarcinoma histology and pathological stage III disease. Because sex was not retained in the final nomogram model, it was not assigned a point value. The nomogram score was calculated according to the four predictors included in the model.

| **Predictor** | **Patient characteristic** | **Approximate nomogram points** |
| --- | --- | --- |
| Age | 69 | 0 |
| Diabetes mellitus | No | 0 |
| Histology | Adenocarcinoma | 100 |
| Pathological stage | Stage III | 80 |
| Total score | — | 180 |
| Estimated postoperative DVT probability | — | 82% |

According to the nomogram, the patient received 0 points for age <70 years, 0 points for absence of diabetes mellitus, approximately 100 points for adenocarcinoma histology, and approximately 80 points for pathological stage III disease. The total nomogram score was therefore approximately 180 points, corresponding to an estimated postoperative DVT probability of approximately 82%.
